# Portage vaginal du streptocoque du groupe B chez la femme enceinte au niveau de la région de Marrakech

**DOI:** 10.11604/pamj.2016.23.107.9047

**Published:** 2016-03-16

**Authors:** Ahlam Bassir, Hanane Dhibou, Majdi Farah, Lharmis Mohamed, Addebous Amal, Souraa Nabila, Aboulfalah Abderahim, Hamid Asmouki, Abderraouf Soummani

**Affiliations:** 1 Service de Gynécologie Obstétrique, Hôpital Mère-Enfant CHU Mohammed VI Marrakech, Maroc; 2 Laboratoire de microbiologie, Hôpital Mère-Enfant CHU Mohammed VI Marrakech, Maroc

**Keywords:** streptocoque B, grossesse, portage vaginal, Group B streptococcal, pregnancy, vaginal colonization

## Abstract

**Introduction:**

Le streptocoque du groupe B est le principal agent impliqué dans les infections materno-fœtales, les septicémies et les méningites du nouveau-né à terme. L'objectif est de déterminer le taux de portage maternel du streptocoque du groupe B (SGB) à terme.

**Méthodes:**

Un prélèvement vaginal a été réalisé de manière prospective chez 275 parturientes lors de l'entrée en salle d'accouchement sur une période de 06 mois.

**Résultats:**

Le taux de portage était de 20,2%. Le portage était variable en fonction de l’âge gestationnel, il constitue 57.5% entre 37 et 38 semaines d'aménorrhée. Aucun des facteurs de risque n'a était statistiquement prédictif du portage maternel du SGB.

**Conclusion:**

Le dépistage doit être réalisé à partir de 37 semaines d'aménorrhée, et comme le portage est intermittent, un prélèvement négatif ne garantirait pas que le portage soit négatif à l'accouchement.

## Abstract (en)

**Introduction:**

Group B streptococcus is the main etiological agent of maternal and fetal infections, sepsis and meningitis in the term newborns. The objective is to determine group B streptococcal (GBS) carriage rate in vagina of term pregnant women.

**Methods:**

Vaginal swab was performed prospectively in 275 pregnant women in labor, prior to entering the delivery room over a period of 6 months.

**Results:**

Colonization rate was 20.2%. The bearing was variable according to the gestational age, it constitutes 57.5% between 37 and 38 weeks of amenorrhoea. None of the risk factors was statistically predictive for maternal GBS colonization.

**Conclusion:**

Screening should be performed from 37 weeks of amenorrhoea and, as colonization is intermittent, a negative swab result does not ensure that GBS colonization is eradicate at birth.

## Introduction

Le streptocoque du groupe B (SGB) ou Streptococcus agalactiae est un germe banal, dont le réservoir humain est digestif et qui est également retrouvé fréquemment au niveau vaginal. Le portage est le plus souvent asymptomatique, mais il peut occasionnellement être responsable d'infections génitales de gravité variable [1]. Il est considéré comme le principal agent impliqué dans les infections materno-fœtales, les septicémies et les méningites du nouveau-né à terme [1, 2]. En raison de l'importance de la colonisation maternelle et du pouvoir pathogène de cette bactérie, des stratégies de dépistage, de prévention et de traitement ont été développées. L'objectif étant d'individualiser les patientes porteuses de streptocoque du groupe B au moment de l'accouchement afin de leur offrir un traitement antibiotique, seul moyen réellement efficace pour prévenir les infections néonatales précoces [3]. L'objectif de notre étude est de déterminer le taux de portage maternel de streptocoque du groupe B à terme et rechercher les éventuels facteurs prédictifs de ce portage. Nous avons réalisé une étude prospective transversale sur une durée de 06 mois, afin d’évaluer la prévalence du portage maternel du SGB à l'entrée en salle de travail, et de rechercher les éventuels facteurs de risque de ce portage.

## Méthodes

Une étude prospective transversale à visée descriptive réalisée sur une durée de 06 mois du juin 2012 au décembre 2012 ayant inclus 257 parturientes. Le choix a été aléatoire au niveau de la salle d'accouchement du centre hospitalier universitaire Mohamed VI de Marrakech. Nous avons exclu toutes les parturientes ayant reçu une antibiothérapie quelque soit son type et sa durée dans les 15 jours précédant. De même toutes les patientes avec une rupture prématurée des membranes avant 37 semaines d'aménorrhée (SA) et celle ayant bénéficié d'une césarienne à froid n'ont pas été incluses dans cette étude. Ainsi, dès l'admission un prélèvement vaginal (au niveau du tiers inférieur sans atteindre le cul-de- sac vaginal) sans spéculum est réalisé. Les échantillons prélevés ont été acheminés au laboratoire de microbiologie dans un délai maximum de trois heures. Au laboratoire, les prélèvements ont été ensemencés dans des milieux de culture prêts à l'emploi à base de gélose au sang contenant un supplément en colistine et en acide nalidixique et incubés à 37 °C pendant 24 heures sous CO2 puis repiqués sur une gélose au sang frais. La recherche des colonies B-hémolytiques et non hémolytiques a était effectuée sur le milieu de culture. Toute colonie bêtahémolytique qui apparaissait en 24-48 heures d'incubation et dont la réaction catalase était négative, a été identifiée par séro-groupage de Lancefield. En cas d'agglutination, le diagnostic de SGB était retenu. Nous avons évalué la sensibilité des isolats du SGB aux antibiotiques par la technique de diffusion en milieu gélosé de Muller-Hinton. Le déroulement du travail et de l'accouchement ont été gérés de manière classique avec prescription d'une antibiothérapie au cours du travail chez les patientes avec un des facteurs de risque suivants: rupture prématurée des membranes de plus de 12 heures et/ou fièvre au cours du travail supérieure à 38°C. Ces femmes recevaient systématiquement 2g d'amoxicilline dès la constatation du symptôme puis 1g toutes les quatre heures jusqu’à l'accouchement. En cas d'allergie à la pénicilline ces patientes recevaient de l’érythromycine à la dose de 1g toutes les huit heures. Les caractéristiques sociodémographiques (âge, niveau socioéconomique, gestité, parité, âge gestationnel), les antécédents gynéco-obstétricaux (antécédents d'interruption volontaire de grossesse, fausse couche spontanée, grossesse extra-utérine, mort f'tale in utero, pyélonéphrite, menace d'accouchement prématuré) et les événements intervenus lors de la grossesse actuelle (grossesse unique ou multiple, diabète gestationnel, pyélonéphrite, menace d'accouchement prématuré) ont été recherchés et consignés sur une fiche de renseignement spécifique. De même les caractéristiques des nouveau-nés (poids de naissance, sexe, score d'Apgar à cinq minutes) ont été recherchées et notées. Le recueil des facteurs de risque était réalisé par l'interrogatoire des parturientes dès l'entrée en salle de travail et complété par un recueil sur le dossier médical juste après l'accouchement pour les paramètres liés au travail et à l'accouchement. Le critère de jugement principal de cette étude était le taux de portage du SGB lors de l'admission en salle d'accouchement.

## Résultats

Parmi les 257 patientes dépistées, 52 avaient un prélèvement positif au SGB ce qui correspond à un taux de portage de 20,2%. L’âge moyen des patientes était de 29,7 avec un écart type de 6,8. L’âge moyen des patientes ayant un portage était de 30,1 avec un écart type de 7,9 et l’âge moyen des patientes saines est 28,7 avec un écart type de 6,8. L’âge gestationnel était entre 37 SA et 38 SA chez 168 patientes (81,9%) (Tableau 1). Parmi les patientes dépistées, l'antécédent d'infection génitale (leucorrhée) a été retrouvé chez 66 patientes non porteuse du streptocoque B (32,2%), et chez 27 patientes porteuse de streptocoque B (51,9%). L'antécédent d'infection néonatale a été retrouvé chez 17 patientes non porteuses du streptocoque groupe B (8,5%) et chez 4 patientes porteuses du streptocoque du groupe B (10,5%). Le taux d'accouchement par voie basse était de 80% chez 159 patientes non porteuses du streptocoque du groupe B et de 76,3% chez 29 patientes porteuses du streptocoque (Tableau 2). Les souches du streptocoque identifié étaient sensibles aux pénicillines avec un taux de 94% (49 patientes).

**Tableau 1:** Le taux de portage du streptocoque B en fonction de l’âge gestationnel

Age gestationnel	nombre	%
34 à 35 SA	7	13,4
35 à 36 SA	4	7,8
36 à 37 SA	10	21
37 à 38 SA	31	57.7
Total	52	99.9

**Tableau 2:** Données cliniques des patientes prélevées

Collège	Nouveaux étudiants	Étudiants du deuxième cycle
Age moyen	28.7 +/- 6.8	30.1+/- 7.9
Gestité		
Primigestes n (%)	101 (49.2)	25 (48.1)
>1 geste	104 (50.7)	27 (51.9)
Age gestationnel		
34-37 SA	37 (18.1)	21 (40.4)
>37 SA	168 (81.9)	31 (59.6)
Antécédent d'infection		
néonatale n (%)	17/199 (8.5)	4/38 (10.5)
leucorrhée n (%)	66 (32.2)	27 (51.9)

## Discussion

Les recommandations concernant le dépistage du SGB sont nombreuses. L'American Academy of pediatrics (AAP), le Collège national des Gynécologues Obstétriciens Français et l'ANAES recommandent le dépistage du SGB chez les patientes asymptomatiques et sans antécédents d'infections materno-fœtales associée à une antibiothérapie per-partum en cas de dépistage positif [1]. L'American College of Obstetrics and Gynecology associé à l'AAP et le Center for Disease Control and Prevention (CDC) ont proposé en 1996, deux options, considérées comme équivalentes, celle du dépistage systématique et celle basée sur la recherche de facteurs de risque justifiant la mise en route de l'antibiothérapie per-partum [4–6]. Dans notre étude, le portage asymptomatique du streptocoque du groupe B chez les parturientes à terme est de 20,2% ce qui est corrélé avec les résultats de la littérature. En France, le nombre de femmes enceintes porteuses du SGB a été à estimé à 10%, ce qui correspond à 75 000 femmes enceintes par an [7], de même une étude récente réalisée à l'hôpital Saint-pierre en 2011 portant sur 17 430 patiente le taux du portage du streptocoque du groupe B était de 16,7% [8]. Au Canada la colonisation maternelle varie entre 11% et 19,5% [9]. En Belgique, dans une population de 1222 patientes, le taux de portage du streptocoque du groupe B est de 23,7% [10]. Aux États-Unis, le portage vaginal asymptomatique du SGB a été estimé à 20 à 30% des femmes enceintes en fin de grossesse [2]. La prévalence du portage génital et anal du streptocoque du groupe B dans les pays en voie de développement est estimée à 9% en Inde, 8% en Asie du Pacifique, 18% en Afrique subsaharienne, 17% en Afrique du Nord et de l'Est, 12% en Amérique centrale ou du Sud [11]. En Tunisie la prévalence a été estimée à 12,92% sur une population de 294 patientes dépistées [12]. Dans une étude prospective réalisé au CHU Hassan II de Fès (Maroc) portant sur une population de 240 parturiente dépistée, le taux de portage du streptocoque du groupe B était de 23,3% [13]. Le portage maternel du Streptocoque du groupe B est inconstant et variable au cours de la grossesse. Dans notre étude, le portage a été estimé à 59.6% entre 37 et 38 semaine d'aménorrhée, alors qu'entre 36 et 37 SA et 34,35 SA il n'a été estimée qu’à 40.4% (Tableau 3). Dans la littérature, il a été démontré que le taux de portage est de 11,4% entre 22 et 26 SA et que 6,4% des patientes avec prélèvement négatif auront un prélèvement positif plus tard dans la grossesse alors que 28,6% des patientes avec prélèvement positif initialement verront disparaître leur portage plus tard [14].

**Tableau 3:** Prévalence du portage du streptocoque du groupe B chez la femme enceinte selon les pays

Pays	Prévalence du SGB
Inde	9
Amérique du Sud	12
Tunisie	12.9
France	16.7
Asie du pacifique	18
Canada	19.5
Maroc, Marrakech	20.2
Maroc, Fès	23.3
Belgique	23.7
Etats-Unis	30

Le taux de portage varie également en fonction des sites prélevés. Dans notre étude, les prélèvements ont été réalisés uniquement au niveau génital. Dans l’étude réalisée à Fès, le portage anal seul a été estimé à 11,7%, alors que le portage vaginal seul a été estimé à 3,3%, et le portage vaginal et anal à 23,3% [13]. Une étude portant sur 370 femmes dépistées entre 35 et 37 SA, 57 étaient colonisées par le SGB. Dans 16 cas (28%) seul le prélèvement anal était positif et dans huit cas (14%) seul le prélèvement vaginal était positif [12]. De même dans une série brésilienne et sur 35 prélèvements positifs (sur 207 femmes dépistées), 15 l’étaient seulement sur le prélèvement anal, soit 43% [15]. Les études réalisées en Amérique du Nord comportent l'association d'un prélèvement rectal systématique expliquant un portage régulièrement supérieur à 18% [10]. En revanche, en France, ce prélèvement est jugé inutile en l'absence d'intérêt démontré en terme d'infections materno-foetales évitées [1]. Par ailleurs, la technique du prélèvement prend une place importante. La colonisation la plus importante de SGB se trouve au niveau du tiers inférieur, ainsi l'utilisation du spéculum masque la face antérieure et postérieure du vagin, réduisant la surface étudié. Cependant, aucune recommandation n'a été émise par les sociétés savantes. Lors du prélèvement, il est recommandé d'insister sur l'importance du balayage de la partie intérieure du vagin jusqu'au vestibule et la vulve comme recommandé par l'ANAES d'où la technique d'introduire l’écouvillon sans spéculum [16]. Le taux de portage est également corrélé à la technique bactériologique employée. Ainsi, un prélèvement vaginal ensemencé sans enrichissement sélectif, tel qu'il est recommandé par l'ANAES, permet de retrouver du SGB chez 10% des gestantes. En revanche un ensemencement avec un enrichissement sélectif augmente le taux de positivité atteignant 15% [17]. Le risque infectieux est proportionnel à la densité du portage. Il a été démontré que le risque de transmission est lié à l'inoculum du SGB présent. Les facteurs étudiés lors de la grossesse au moment du dépistage (l’âge, la parité, la gestité, antécédents gynéco-obstétricaux, menace d'accouchement prématuré) n'ont pas montré un véritable intérêt comme facteurs associés de façon significative au portage de SGB. Bien que la littérature concernant le dépistage SGB soit abondante, rares sont les études qui se sont penchée spécifiquement sur les facteurs de risque du portage maternel de ce germe. L'antibioprophylaxie pendant le travail doit être brève, intense, dose de charge et voie intraveineuse, avec des molécules à spectre étroit [3]. L'ANAES [1] et l'American Academy of Pediatrics [5] recommandent de débuter précocement l'antibiothérapie au cours du travail car son efficacité n'est optimale qu’à partir de la deuxième injection. Il faudrait un délai d'au moins 4 heures pour obtenir un taux d'ampicilline efficace dans le liquide amniotique et réduire ainsi efficacement la transmission. L'antibiothérapie préventive locale pendant la grossesse ne diminue pas le taux de portage à l'accouchement.

## Conclusion

Les infections néonatales sont une cause de mortalité et de morbidité néonatales, notamment les infections à streptocoques du groupe B, qui en représentent 30 à 50%. Un dépistage correct et une conduite à tenir appropriée paraissent donc nécessaires. Mais ce dépistage, qui a montré son efficacité, a également des limites, qui doivent être discutées. Au terme de cette étude et en absence de vaccination efficace contre ce germe, la stratégie actuelle de prévention consiste non pas à réduire la colonisation mais à limiter le risque de transmission materno-fœtale.

### Etat des connaissance sur le sujet

- Le streptocoque du groupe B est le principal agent impliqué dans les infections materno fœtales, les septicémies et les méningites du nouveau-né à terme.
- En raison de l'importance de la colonisation maternelle et du pouvoir pathogène de cette bactérie, des stratégies de dépistage, de prévention et de traitement ont été développées.

### Contribution de notre étude a la connaissance

- Déterminer le taux de portage maternel de streptocoque du groupe B à terme dans notre région et rechercher les éventuels facteurs prédictifs de ce portage.
- Avoir plus de données épidémiologiques sur le portage du streptocoque du groupe B au Maroc.

## References

[1] (2001). Prévention anténatale du risque infectieux bactérien néonatal précoce. Recommandations pour la pratique clinique. Recommandations pour la pratique clinique. Agence nationale de l'Accréditation et de l’Évaluation en Santé.

[2] Gibbs RS, Schrag S, Schuchat A (2004). Perinatal infections due to group B streptococci. Obstet Gynecol..

[3] Jaureguy F, Carton M, Teboul J, Butel MJ, Panel P, Ghnassia JC (2003). Facteurs de risque et stratégie de dépistage de la colonisation par le streptocoque du groupe B chez la femme enceinte: résultats d'une étude prospective. J Gynecol Obstet Biol Reprod (Paris)..

[4] Blanc B, Blond MH, Chaix C, Goffinet F, Guillaume S, Judlin P (1997). Les infections cervico-vaginales au cours de la grossesse. Collège National des Gynécologues et Obstétriciens Français.

[5] American Academy of Pediatrics, Comittee on infection Diseases and Comittee on Fetus and newborn (1992). Guidelines for prevention of group B Streptococcal infection by chemoprophylaxis. Pediatrics.

[6] Centers for Disease Control and Prevention (1996). Prevention of perinatal group B streptococcal disease: a public health perspective. MMWR Recomm Rep.

[7] (2003). Agence nationale d'accréditation et d’évaluation en santé. Prévention anténatale du risque infectieux bactérien néonatal précoce. J Gynecol Obstet Biol Reprod (Paris).

[8] Dahan-Saala J, Rardin PGe, Robillard PY (2011). Déterminants de la colonisation maternelle a streptocoque B et facteurs associés à sa transmission verticale périnatale: étude cas-témoins. Gynécologie Obstétrique et Fertilité..

[9] Money DM, Dobson S, Canadian Paediatric Society, Infectious Diseases Commitee (2004). The prevention of early-onset neonatal group B streptococcal disease. J Obstet Gynaecol Can.

[10] Lorquet S, Melin P, Minon JM, Carpentier M, Gerday C, Rigo J (2005). Le streptocoque du groupe B en clinique anténatale et en salle de travail: un problème d'attitude systématique. J Gynecol Obstet Biol Reprod (Paris)..

[11] Stoll BJ, Schuchat A (1998). Maternal carriage of group B streptococci in developing countries. Pediatr Infect Dis J..

[12] Jerbi M, Hidar S, Hannachi N (2007). Facteurs de risque du portage du streptocoque du groupe B chez la femme enceinte à terme: étude prospective à propos de 294 cas. Gynecol Obstet Fertil..

[13] Mahmoud M, Yahyaoui G, Benseddik N (2011). Dépistage de streptocoque du groupe B au cours du troisième trimestre de grossesse au CHU Hassan II de Fès. Revue Tunisienne d'Infectiologie.

[14] Kubota T (1998). Relationship between maternal group B streptococcal colonization and pregnancy outcome. Obstet Gynecol..

[15] El Beitune P, Duarte G, Maffei CM, Quintana SM, De Sa Rosa E, Silva AC (2006). Group B Streptococcus carriers among HIV-1 infected pregnant women: Prevalence and risk factors. Eur J Obstet Gynecol Reprod Biol..

[16] Loulergue J, Couhé C, Grasmick C (2004). Sensibilité aux antibiotiques des souches de streptocoque du groupe B de portage vaginal isolées en France, 2003. BEH..

[17] Benitz WE, Gould JB, Druzin ML (1999). Preventing early-onset group B streptococcal sepsis: strategy development using decision analysis. Pediatrics..

